# Blood loss of total knee arthroplasty in osteoarthritis: an analysis of influential factors

**DOI:** 10.1186/s13018-018-1038-0

**Published:** 2018-12-22

**Authors:** Yong Hu, Qiang Li, Bao-Gang Wei, Xian-Sen Zhang, Tahsin Tarik Torsha, Jun Xiao, Zhan-Jun Shi

**Affiliations:** 1 Department of Orthopaedic Surgery, Nanfang Hospital, Southern Medical University, Guangzhou, 510515 Guangdong Province China; 2 Department of Orthopaedic Surgery, Chinese Traditional Medicine Hospital of Huaihua City, Huaihua, 418000 Hunan Province China; 3 Department of Orthopaedic Surgery, Inner Mongolia People’s Hospital, Hohhot, 100017 Inner Mongolia China; 4 Department of Orthopedic Surgery, The Third People’s Hospital of Dongguan City, Dongguan, 523326 Guangdong Province China; 5 Southern Medical University, Guangzhou, 510515 Guangdong Province China

**Keywords:** Total knee arthroplasty, Blood loss, Influential factor

## Abstract

**Background:**

Total knee arthroplasty is regarded as the most effective treatment for severe knee osteoarthritis. The influential factors of blood loss in total knee arthroplasty remain controversial. The study aims to explore the influential factors of blood loss in total knee arthroplasty comprehensively.

**Material and methods:**

Three hundred and four osteoarthritis patients undergoing unilateral primary total knee arthroplasty were enrolled. Demographic characteristics, laboratory results, surgical protocol, and hemostatic and anticoagulation drugs were collected. Estimation of blood loss was calculated using the Gross equation. Multivariable stepwise linear regression analysis was performed to find out the influential factors.

**Results:**

Total blood loss reached the biggest volume (1346 ± 671 mL) in the post-operative third day. Hidden blood loss reached 465 ± 358 mL. Gender, tranexamic acid, prosthesis type, and drainage were proven to be positively correlated with the total blood loss (all *P* < 0.05). Male appeared to suffer more surgical blood loss than female. Posterior cruciate stabilizing prosthesis might lead to more surgical blood loss than posterior cruciate retaining prosthesis. Tranexamic acid could effectively reduce total blood loss while drainage might increase bleeding. Gender and anticoagulation drugs were correlated with hidden blood loss (both *P* < 0.05). Low molecular weight heparin resulted in less hidden blood loss than rivaroxaban.

**Conclusions:**

Posterior cruciate retaining prosthesis and topical use of tranexamic acid were preferred to reduce total blood loss. Drainage was not recommended due to the risk of increasing bleeding. Low molecular weight heparin was recommended to prevent venous thrombosis.

## Background

Symptomatic knee osteoarthritis (OA) is highly prevalent amongst older people worldwide, and the morbidity even reached 30%, especially in rural regions [1]. Total knee arthroplasty (TKA) is regarded as the most effective treatment for severe knee OA, with rates of excellent or good long-term outcomes reaching 98% [2–4]. Despite significant improvement in surgical techniques [5], the large blood loss after TKA remains a concern of surgeons. The total blood loss even reached 1500 mL in average [5–8], which also led to high transfusion rate. Up to 70% of patients who undergo total joint arthroplasty received blood transfusion [9]. Allogeneic blood transfusion might increase the risk of longer hospital stays, infectious disease transmission, immunologic reactions, hemolytic and anaphylactic reactions, and increased mortality [10].

Identifying influential factors of surgical blood loss is an important step toward establishing an effective blood management strategy and further reducing the need for perioperative blood transfusion. However, the existing results of influential factors in OA patients undergoing TKA remain controversial. Cushner and Friedman found that gender of the patients was positively related to blood loss, but excluded the correlation of age, diagnosis, and operative time [11]. Another research conducted by Lošťák et al. proposed different opinion, and they declared that the characteristics affecting the amount of blood loss in TKA were BMI, preoperative platelet count, and INR [7]. Mesa-Ramos et al. denied the correlation between age, sex, BMI, and blood loss in TKA [12]. Tranexamic acid (TXA) [8, 13], tourniquet [14], and anticoagulation drugs [15] were also proven to be positively associated with blood loss in TKA. But some potential risk factors, such as coagulation system, electrolyte disturbance, prosthesis type, and material of plugging the femoral hole, are lack of further study.

In the present study, 304 cases undergoing unilateral TKA in our department from the year 2011 to 2016 were enrolled in the study. Excluding from the influence of diagnosis, we only included patients diagnosed as having knee osteoarthritis. Gross equation was adopted to estimate the volume of surgical blood loss. Multivariable stepwise linear regression analysis was performed to explore the influential factors, in which we included as many risk factors as possible. The potential risk factors include gender, BMI, coagulation indexes, electrolyte, surgical protocol, and hemostatic and anticoagulation drugs. The study aims to comprehensively explore the exact factors influencing blood loss in total knee arthroplasty surgery.

## Methods

### Patients

Patients who were diagnosed with knee OA and underwent unilateral TKA from January 2011 to June 2016 were included in the study. Those with the following conditions were excluded, including rheumatoid arthritis, ankylosing spondylitis, Kashin Beck disease, active infection, tumor, severe cerebrovascular disease, liver disease with severe abnormal liver function, and hematologic diseases like coagulation dysfunction (such as hemophilia) and thrombocytopenia. In addition, patients with long-term use of anticoagulant drugs were also excluded. For hypertensive patients, the preoperative blood pressure was controlled to beneath 140/90 mmHg.

Finally, 304 patients consisting of 61 males and 243 females were included in the study. The average age was 56.8 years, ranging from 42 to 80. The average BMI was 26.46 kg/m^2^, which was defined as overweight. The different protocol of the patients was described in Table 1, and the laboratory test results were shown in Table 2.

**Table 1:** Patients’ characters

	Variables	Value
Gender	Male	61
	Female	243
Prosthesis type	Posterior cruciate stabilizing	204
	Posterior cruciate retaining	100
Anticoagulation	Low molecular weight heparin	129
	Rivaroxaban	175
Hemocoagulase agkistrodon	Non-utilization	37
	Utilization	267
Material of filling the location hole of femurs	Nothing	124
	Cement	98
	Bone	82
Use of TXA	Topical application	63
	Combined application	54
	Non-application	122
	Intravenous application	65
Drainage	Non-utilization	202
	Utilization	102

**Table 2:** Assessment of the patients

	Mean ± SD
BMI (kg/m^2^)	26.46 ± 3.495
Preoperative APTT (s)	28.87 ± 4.378
Preoperative Fbgc (g/L)	3.24 ± 1.058
Preoperative Hct	0.39 ± 0.037
Preoperative kalium (mmol/L)	4.02 ± 0.331
Preoperative calcium (mmol/L)	3.19 ± 9.98
Preoperative magnesium (mmol/L)	0.88 ± 0.110
Operation time (min)	109.72 ± 25.88
Preoperative PT (s)	11.49 ± 1.144
Preoperative TT (s)	17.41 ± 1.896
Preoperative Hb (g/L)	12.797 ± 1.4347
Preoperative ALB (g/L)	38.91 ± 3.688
Preoperative natrium (mmol/L)	141.87 ± 2.941
Preoperative phosphorus (mmol/L)	1.20 ± 0.177
Age (years)	66.15 ± 7.58

### Surgical indication, technique, and perioperative management

Severe pain affecting daily activity, dysfunction of joint motion, and radiography evidence that narrowing joint space with cystic change of subchondral bone, bone sclerosis and formation of osteophyma, were the main indications for OA patients to receive TKA after invalid conservative treatment.

Standard surgical methods were adopted for all patients (median knee incision, with incision length ranging from 10 to 15 cm), and all the surgeries were performed in the supine position by the same experienced team. General anesthesia or spinal anesthesia was performed. Tourniquet was commonly used during the operation, and the pressure was set in 60 kPa. Cephalosporins were routinely applied for 24 h after operation to prevent infection. Two kinds of prostheses included posterior cruciate retaining (CR) prosthesis and posterior cruciate stabilizing (PS) prosthesis. The material of plugging the location hole of femur included cement and bone. Autologous re-transfusion drainage regimen was applied in the study, which had beneficial effects of higher post-operative hemoglobin and lower allogenic transfusion rate [16, 17]. The drainage tube was located in the proximal lateral incision into the joint cavity, which was connected to an autologous re-transfusion drainage device (ConstaVac TM CBC II, Stryker, USA) that worked under the minimum level of negative pressure. The reinfusion process was completed within 6 h from the beginning of collecting drainage blood for each case. Tranexamic acid (TXA) with different usage method was applied, which included topical use, intravenous use, and combined use. Hemocoagulase agkistrodon (HCA) was also applied for reducing bleeding in some cases. Low molecular weight heparin (LMWH) or rivaroxaban was used in 12 h post-operatively to prevent deep venous thrombosis (DVT).

Physical exercise and continuous passive motion exercise device (CPM) were applied on post-operative day 1 until discharge. Physical exercises were performed twice daily according to standard guidelines [18, 19], including isometric quadriceps exercise, active knee flexion and extension exercise, ankle pump exercise, and straight leg raising exercise. Referring to the studies of Pope et al. [19] and Goletz et al. [20], all patients received CPM with an initial range of 0 to 40° and 10 to 15° of increments daily. All patients received three CPM sessions per day (each lasting 2 h) in a supine position with the head of the bed 30 to 60° angle of inclination [21].

### Collecting data

Age, sex, height, and weight were recorded. Both preoperative hemoglobin (Hb) and hematocrit (Hct) and post-operative results in the first, third, and fifth days after operation were collected. Preoperative laboratory test results including prothrombin time (PT), activated partial thromboplastin time (APTT), thrombin time (TT), fibrinogen concentration (Fbgc), albumin (ALB), kalium, natrium, calcium, phosphorus, and magnesium were collected. Prosthesis type, material of plugging the location hole of femur, administration of TXA, HCA, and anticoagulant drug were also recorded.

### Estimation of blood loss

Total blood loss was estimated with Gross equation [22]. The actual blood loss could be acquired by subtracting the autologous blood transfusion and the allogenetic blood transfusion. The specific calculation method for the Gross equation: total blood loss = patient blood volume (PBV)^2^ × (preoperative Hct – post-operative Hct)/(preoperative Hct + post-operative Hct). In which PBV = k1 × [height (m)]^3^ + k2 × body weight (kg) + k3. For males, k1 = 0.3669, k2 = 0.0329, and k3 = 0.6041; for females, k1 = 0.3561, k2 = 0.033 08, and k3 = 0.1833. The total blood losses of the first, third, and fifth days after the operation were calculated. In addition, the increased blood losses between the first day and third day after operation were calculated, which could be considered as hidden blood loss after operation.

### Transfusion management

Both allogenic transfusion and autologous transfusion were employed during the operation and post-operatively, which was based on the condition of patients. The post-operative draining blood was collected for autologous transfusion that was performed 6 h after operation with an autologous negative pressure drainage device, ConstaVac TM CBC II (Stryker, USA). The allogenic transfusion triggers were based on the British guideline [23] and clinical judgment. On the one hand, the traditional view on transfusion triggers was Hb < 70 g/L and hematocrit < 25%. Hb < 80 g/L was a transfusion trigger for patients with cardiovascular and respiratory problems or patients aged > 65 years. On the other hand, clinical manifestations presenting by that were considered as acute anemia, such as a drop in blood pressure (< 90/60 mmHg), dizziness, pale lips, weakness, and shortness of breath, were also regarded as transfusion trigger.

### Statistical analysis

Twenty-two kinds of factors were divided into quantitative and quantitative variables. Multivariable stepwise linear regression was used to find out the impact factors of blood loss. In consideration of the peak of post-operative blood loss, the blood loss in the third day after TKA was chosen as the dependent variable. The continuous blood loss between the first day and third day after operation also attracted our attention. That was also set as a dependent variable.

Fifteen quantitative variables were included: BMI, PT, APTT, TT, Fbgc, preoperative Hb, preoperative HCT, ALB, kalium, natrium, calcium, phosphorus, magnesium, age, and operation time. Seven qualitative variables were included in the multiple linear regression analysis. For the gender, the male was set as “1” and female was “0.” For the different prosthesis type, “CR” was set as “0” and “PS” was set as “1.” For the utilization of drainage, “without drainage” was set as “0” and “with drainage” was set as “1.” For the material of plugging the location hole of femurs, “nothing” was set as “0,” “cement” was set as “1,” and “bone” was set as “−1.” For the use of tranexamic acid, four subgroups were set. “Not use” was set as “1”, and the rest was a dummy variable. Same method was used for “topical application,” “intravenous application,” and “combined application.” For the use of hemocoagulase agkistrodon, “not use” was set as “0” and “use” was set as “1.” For the application of anticoagulation drugs, “LMWH” was set as “1” and “Rivaroxaban” was set as “0.”

## Results

### Estimation of blood loss and transfusion

The total blood loss in the first, third, and fifth days after TKA and the increased blood loss volume were described in Table 3. In the third day after the operation, the average blood loss reached the biggest volume and it was 1346 ± 671 mL. Compared to the blood loss volume in the first day after operation, in an average, 465 ± 358 mL blood loss was found more in the third day. However, averagely 750 ± 392 mL blood loss was found in the third day less than that in the fifth day after TKA.

**Table 3:** Surgical blood loss

	First day	Third day	Fifth day	First to third days	Third to fifth days
	Mean ± SD	Mean ± SD	Mean ± SD	Mean ± SD	Mean ± SD
Volume (mL)	880 ± 560	1346 ± 671	595 ± 637	465 ± 358	− 750 ± 392

More than half of the patients (51.55%) have received transfusion within 3 days after operation, including allogenic transfusion, autologous transfusion, and both. The allogenic transfusion rate reached 35.09%, and the average volume was 0.9 u (0~6 u). 31.68% of the patients received autologous transfusion that reached 169.09 mL in average, of which 49 patients received allogenic transfusion as well. No complications associated with transfusion were found in the study.

### Multivariable stepwise linear regression analysis

**Fig. 1:**
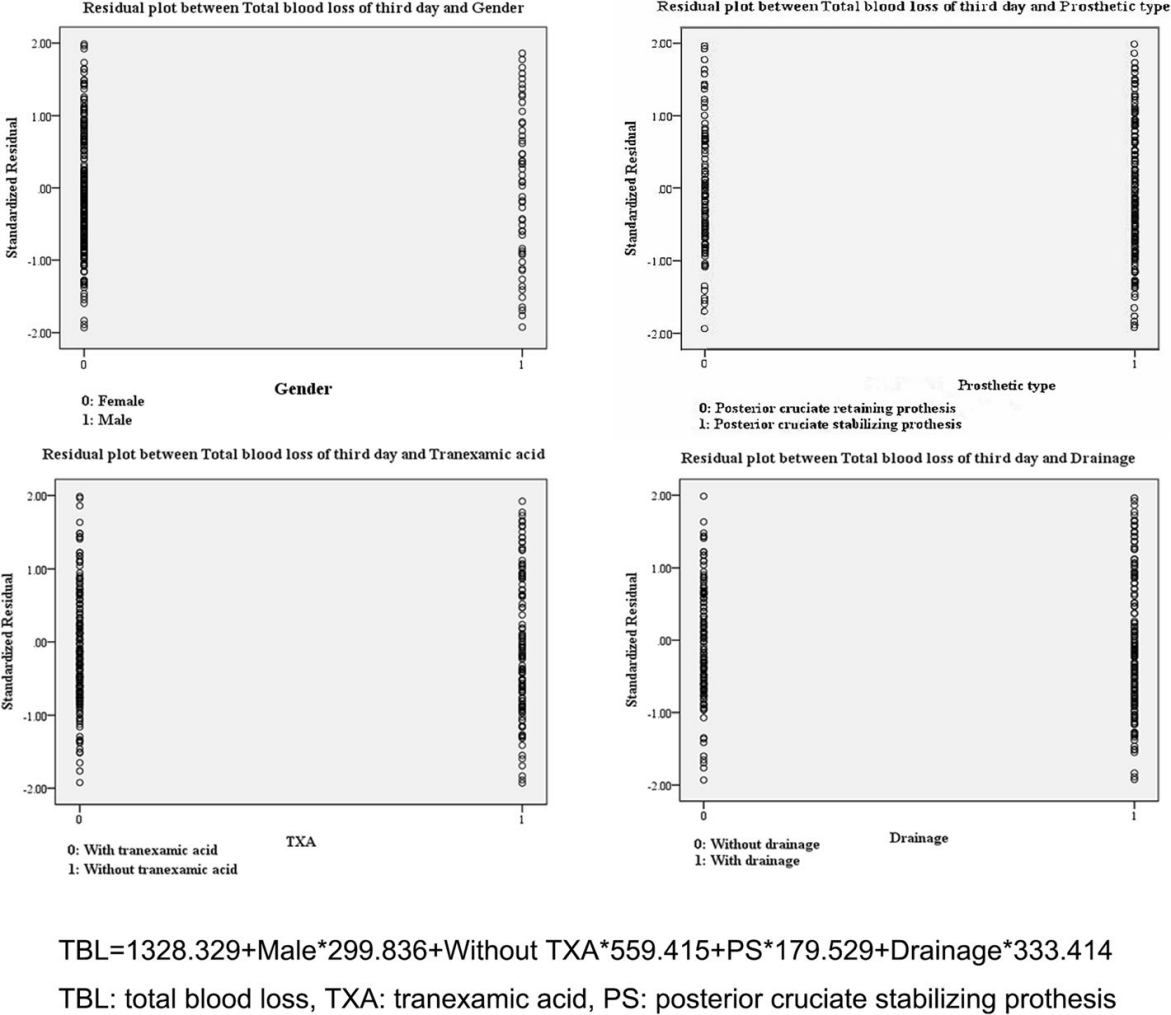
Residual plots between total blood loss and each meaningful factor. The regression model well matches the data

The result of the multivariable stepwise linear regression analysis showed that gender (*P* < 0.001), without TXA (*P* < 0.001), drainage (*P* = 0.019), and prosthetic type (*P* < 0.001) were positively correlated with total blood loss of TKA, but the rest of the potential risk factors presented a negative result. Male appeared to suffer more surgical blood loss than female. PS prosthesis might lead to more surgical blood loss than CR prosthesis. Tranexamic acid could effectively reduce total blood loss while drainage might increase bleeding. However, different strategies of using TXA showed no effect on blood loss. The regression model was shown in Fig. 1.

**Fig. 2:**
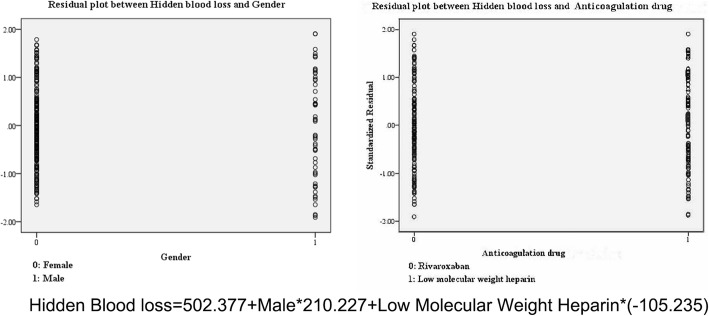
Residual plots between hidden blood loss and each meaningful factor. The regression model well matches the data

Another result showed that gender (*P* < 0.001) and different anticoagulation drugs (*P* = 0.009) were correlated with the hidden blood loss. Similarly, more blood loss was found in the male. Patients who received rivaroxaban suffered more post-operative bleeding. The regression model was shown in Fig. 2.

## Discussion

In the present study, we found that total blood loss was 1346 mL in OA patients undergoing TKA. It was almost 30% of the total blood volume. Consistently, other scholars reported an approximate number. Sehat et al. found that the average blood loss of knee resurfacing arthroplasty was 1471 mL [6]. Digas et al. reported that the total blood loss was 1455 mL for patients with OA undergoing primary TKA. Despite rational use of TXA, the blood loss remained a large volume and it was 1044 mL [8]. Another study reported by Sizer et al. declared that the blood loss of the elderly (> 75 years old) after the primary knee replacement even reached 1741 mL [5].

High transfusion rate was present with the large blood loss eventually, which might influence the post-operative rehabilitation. Astonishingly, Stanworth and other scholars found that the allogeneic blood transfusion rate was as high as 21–70% for patients undergoing TKA [24, 25]. More than half of the patients had received transfusion in our study, of which 68% of the patients needed an allogenic transfusion. Though no complication associated with transfusion was found in the study, the risk of associated complication was reported potentially increased, including extended hospital stays, infectious disease, immune response, and cardiopulmonary overload and reaction of hemolytic [24–26]. Effective steps should be launched to reduce the blood loss and transfusion. It is the primary job to find out the exact influential factors.

Multivariable stepwise linear regression analysis revealed that gender, TXA, drainage, and prosthetic type were correlated with total blood loss of TKA. But we found no correlation with age, BMI, operation time, electrolyte, and coagulation test indexes. Notably, gender difference could affect surgical blood loss including total blood loss and hidden blood loss, with greater amount in men compared with women. The result also received agreement from many scholars [11, 26, 27] who also proved that male had a high risk of suffering more blood loss than female in TKA.

Apart from gender, we also found that different prosthesis type and drainage played a role in influencing blood loss in TKA. The main source of bleeding might be from the venous sinus and the trimmed bone. In the authors’ opinion, PS prosthesis with intercondylar bone osteotomy was possible to suffer more blood loss than CR prosthesis since more bone was cut. Mahringer et al. [28] and Scott et al. [29] announced the same result. The results could possibly be explained by the additional preparation of the femoral box PS TKA for the cam-post mechanism, which resulted in more femoral cancellous bone surface being exposed [28]. However, the materials of plugging the femur had an impact on total blood loss due to the continuous blood loss from venous sinuses of newly appearing cancellous bone. In addition, drainage might release the compression of hemorrhage and exudation, which could reduce the bleeding through oppressing the venous sinus and capillary. Parkers and Roberts indicated that drainage was not recommended for orthopedic surgeons, because no difference was found in the incidence of wound infection, hematoma, or dehiscence between with-drainage and without-drainage. But blood transfusion was required more frequently in those who received drains [30]. The more transfusion also reflected the more blood loss associated with drainage, which supported our results.

The balance between hemostasis and anticoagulation is always a challenge to orthopedic surgeons. With adequate strategy of using hemostasis and anticoagulation, it might reduce blood loss but not increase the risk of thrombus. The result showed that both TXA and anticoagulation drugs including LMWH and rivaroxaban might influence the blood loss.

Regarding the best method of applying TXA, it remains a controversy. Chareancholvanich and co-worker stated that an intravenous injection was the best method for rapidly increasing and maintaining the therapeutic concentration of TXA for patient undergoing TKA [31]. Digas et al. found that intra-articular administration of TXA seemed to be more effective in terms of reducing drained blood loss and transfusion frequency [8]. Another study reported by Li et al. proposed that the combined use of intravenous and topical TXA in patients with total knee arthroplasty was associated with significantly reduced total blood loss, transfusion requirements, post-operative hemoglobin decline, and length of stay compared to single application alone [32]. However, many scholars showed the agreement on our result. They discovered that topical TXA was equally effective compared with intravenous TXA in reducing blood loss and transfusion rate following TKA [33–35].

Therefore, we suggested that topical use of TXA might be the preferential option for patients undergoing TKA, because intravenous application might potentially result in higher risk of complication occurrence than topical application. TXA is an inhibitor of fibrinolysis that acts by blocking the lysine-binding site of plasminogen to fibrin and prevents the degradation of fibrin. Patients utilizing TXA were risky to suffer phlebothrombosis as they have to stay in bed after TKA for a long time, which should raise concern. Wu et al. found that the intravenous use of TXA presents higher deep venous thrombosis (DVT) than the control group, and the incidence reached 6.3% while it was 3.8% with topical use [36]. In addition, Aydin and co-worker proposed other benefits of intra-articular injection of TXA apart from reducing blood loss, which suggested administration of intra-articular TXA significantly shortening the post-operative length of hospital stay and reducing the total hospital costs [37].

The continuous blood loss after operation, or named hidden blood loss, is also a problem that cannot be neglected. The hidden blood loss reached 465 mL in our study. Some scholars suggested that hidden blood loss might be correlated with hemolysis, blood infiltration into tissue compartments, and the use of anticoagulant drugs [38, 39]. To prevent DVT, anticoagulant drugs were irreplaceable for knee replacement. Mainly LMWH and rivaroxaban were the preferred one. Rivaroxaban is an oral anticoagulant that directly inhibits the activity of factor Xa. Carrother et al. found that rivaroxaban can significantly reduce the incidence of DVT [40]. Fuji et al. further proved that the Xa inhibitors have superior efficacy compared with enoxaparin for the prevention of venous thromboembolism in patients following TKA with comparable bleeding [41, 42]. However, Li et al. presented the different conclusion that rivaroxaban did not increase the risk of hidden blood loss but LMWH increased the risk of dominant blood loss [15]. In contrast, we found that LMWH might result in less hidden blood loss while rivaroxaban potentially increases the bleeding. Similarly, Wang et al. [43] and Zou et al. [44] showed the same results.

Different from rivaroxaban that inhibits clotting factor Xa, the major anticoagulant effect of heparin is through its interaction with antithrombin-III (AT-III). This interaction markedly accelerates the ability of AT-III to inactivate the coagulation enzymes thrombin, factor Xa, and factor IXa. Moreover, LMWH, the fragments of standard commercial grade heparin produced by either chemical or enzymatic depolymerization, have reduced ability to catalyze the inactivation of thrombin relative to their ability to inhibit factor Xa because the inactivation of thrombin by heparin is critically dependent on molecular size [45]. Researches of platelet function [46] and vascular permeability [47] have provided plausible explanations for the reduced experimental bleeding observed with LMWH. Higher evidence quality research, a random control cohort study related to the influence of rivaroxaban and LMWH on hidden blood loss, needs to further carry out.

## Conclusions

The total blood loss reached 1346 ± 671 mL in the third day after TKA. The potential influential factors of total blood loss included gender, TXA, prosthesis type, and drainage. Male was considered to suffer more surgical blood loss than female. Perioperative blood management should be enhanced in male patients. Posterior cruciate retaining (CR) prosthesis might be superior in reducing total blood loss of TKA. Drainage was not recommended due to the risk of increasing bleeding. Tranexamic acid was proven to effectively reduce total blood loss, and the topical use was preferred. The hidden blood loss was 465 ± 358 mL, and its impact factors mainly included gender and the different use of anticoagulation. As for the selection to prevent DVT between the low molecular heparin and rivaroxaban, the former was recommended since it might lead to less hidden blood loss.

## Abbreviations

ALB: Albumin
APTT: Activated partial thromboplastin time
AT-III: Antithrombin-III
BMI: Body mass index
CPM: Continuous passive motion exercise device
CR: Posterior cruciate retaining
CRP: C-reactive protein
DVT: Deep venous thrombosis
Fbgc: Fibrinogen concentration
Hb: Hemoglobin
HCA: Hemocoagulase agkistrodon injection
Hct: Hematocrit
LMWH: Low molecular weight heparin
OA: Osteoarthritis
Plt: Platelet
PS: Posterior cruciate stabilizing
PT: Prothrombin time
PT%: Prothrombin activity
PT-INR: Prothrombin time international ratio
TKA: Total knee arthroplasty
TT: Thrombin time
TXA: Tranexamic acid injection
